# A endometrial malignant tumor with yolk sac tumor component: a case report and literature review

**DOI:** 10.3389/fonc.2025.1551266

**Published:** 2025-09-25

**Authors:** Zicheng Cui, Yuping Shan, Huijun Chu, Aiping Chen

**Affiliations:** ^1^ Medical College, Qingdao University, Qingdao, China; ^2^ Department of Obstetrics and Gynecology, The Affiliated Hospital of Qingdao University, Qingdao, China

**Keywords:** yolk sac tumor, endometrial malignancy, primary endometrial yolk sac tumor, AFP, GPC-3, SALL4

## Abstract

**Background:**

Primary endometrial yolk sac tumor (YST), as an extragonadal germ cell tumor, is an exceedingly rare malignancy characterized by high aggressiveness and poor prognosis. Currently, there is no standardized and effective treatment.

**Case report:**

We report a case of a 71-year-old woman with primary mixed endometrial yolk sac tumor. She presented with postmenopausal vaginal bleeding. PET CT imaging suggested a uterine malignancy. Serum levels of AFP, CEA, and CA19–9 were elevated. Diagnostic endometrial biopsy pathology showed adenocarcinoma, suggestive of endometrioid carcinoma. The patient underwent laparoscopic extra-fascial total hysterectomy, bilateral salpingo-oophorectomy, right pelvic lymphadenectomy, and para-aortic lymph node biopsy. Postoperative pathology revealed mixed endometrioid carcinoma, within which a component exhibited features consistent with differentiation towards yolk sac tumor (YST). Immunohistochemistry showed focal AFP positivity (+), SALL4 positivity (+), and GPC3 positivity (+). Postoperatively, she received three cycles of BEP chemotherapy (bleomycin, etoposide, cisplatin), followed by concurrent chemoradiotherapy. Subsequently, she received one cycle of PEB chemotherapy. One month after completing chemotherapy, a PET-CT scan revealed a new lesion in the omentum, indicating disease recurrence. The patient opted for observation. On April 7, 2025, a follow-up chest CT showed multiple small pulmonary nodules, suggestive of metastases. Repeat AFP levels remained within the normal range. She is currently receiving combination therapy with paclitaxel, carboplatin, and pembrolizumab.

**Conclusion:**

Primary endometrial YST is an exceptionally rare malignant germ cell tumor. Early symptoms frequently include vaginal bleeding and abdominal pain, often accompanied by elevated serum AFP levels. Immunohistochemical markers such as AFP, GPC-3, SALL-4, and Villin are valuable for differentiating it from other entities. Advanced FIGO stage and age over 50 years were significantly associated with a higher likelihood of recurrence or death in our case and the available literature. Compared to pure endometrial YST, mixed endometrial YST occurs at an older age and appears to carry a worse prognosis. The currently most treatment for YST is radical surgical resection followed by adjuvant chemotherapy, typically the BEP regimen. The role of radiotherapy in improving outcomes requires further investigation.

## Introduction

Yolk sac tumor (YST) is the third most common malignant germ cell tumor, typically occurring in the gonads of young individuals (1). However, it can also arise in extragonadal sites such as the anterior mediastinum (2), retroperitoneum (3), sacrococcygeal region, and pineal gland. Mixed germ cell tumors (MGCTs), characterized by the presence of two or more malignant, primitive, or germ cell components, constitute approximately 8% of malignant germ cell tumors. The combination of YST and dysgerminoma represents the most frequent MGCT subtype (4).In female patients, extragonadal YSTs account for about 20% of all YSTs (5). Primary endometrial YSTs are even rarer. The first case of a primary endometrial endodermal sinus tumor (EST, synonymous with YST) was described by Pileri in 1980 (6). Subsequently, similar cases were reported sporadically. In 1996, Shokeir MO reported the first case of a malignant mixed Müllerian tumor (MMMT) of the uterus containing a YST component (7), representing the initial documented case of a mixed primary endometrial YST.To date, only 39 cases of primary endometrial YST have been reported in the literature. Among these, 21 cases were pure primary endometrial YSTs, while 18 cases were mixed primary endometrial YSTs. Ravishankar et al. proposed that these endometrial YSTs admixed with other somatic malignancies represent somatic-type YSTs (SDYSTs) derived from somatic malignancies (8). They typically occur in postmenopausal women and are associated with higher aggressiveness and poorer prognosis (8). Herein, we share a case of mixed primary endometrial yolk sac tumor.

## Case presentation

**Figure 1:**
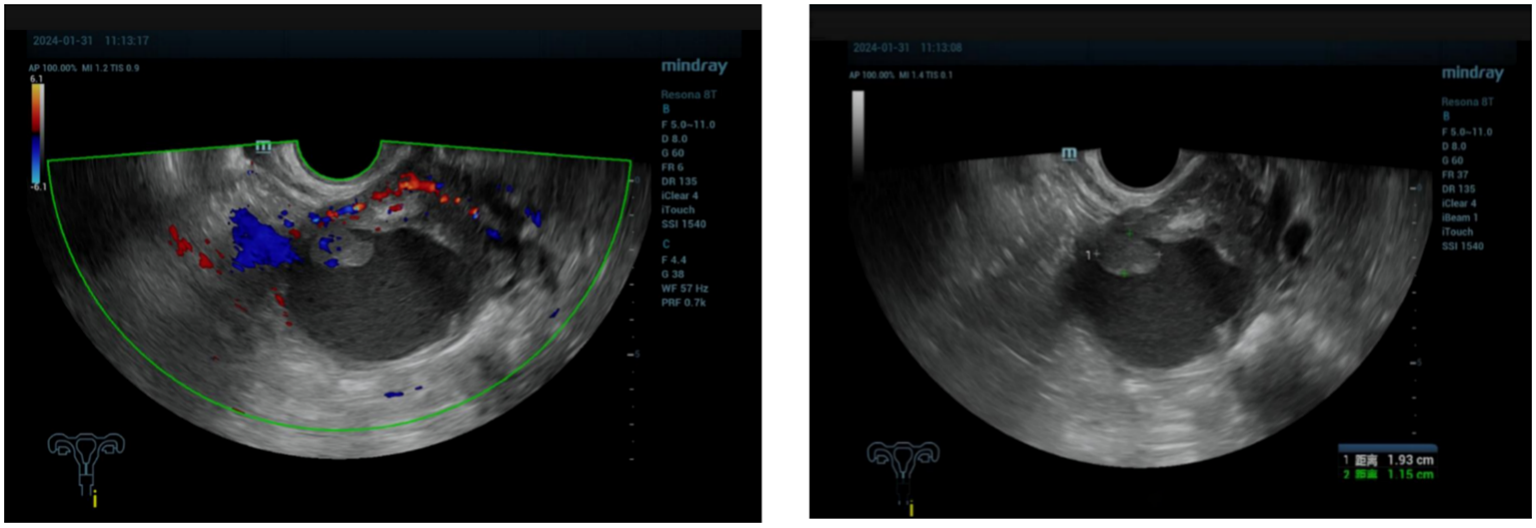
The ultrasound showed enhanced echo in the lower segment of the uterus, ranging from 5.6×4.8×3.8cm, considering endometrial malignancy.

**Figure 2:**
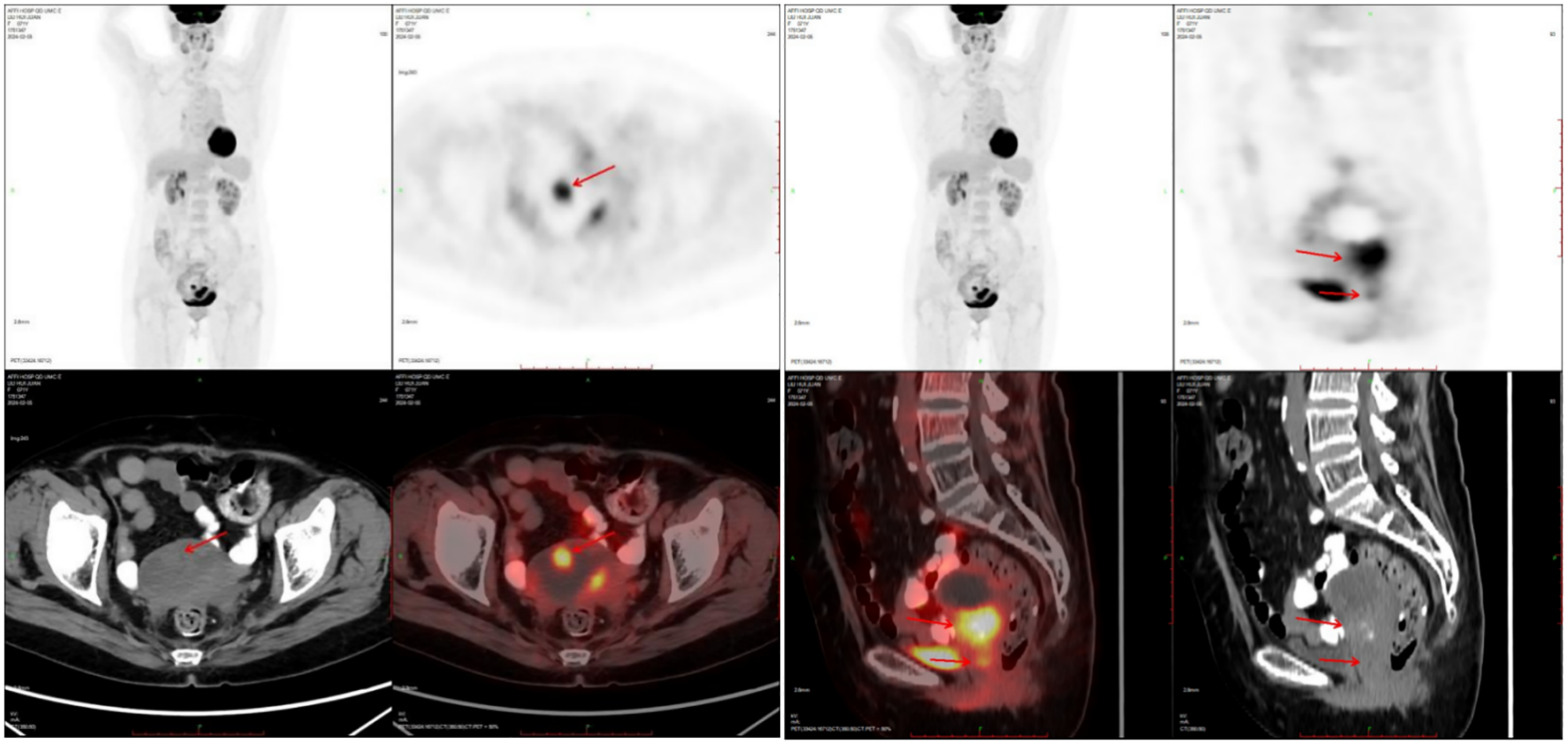
PET-CT showed nodules of soft tissue density at the base of the uterine body, protruding intrauterine, with increased metabolism, which was consistent with malignant uterine tumors.

A 71-year-old postmenopausal woman presented with scant dark-red vaginal bleeding. She sought medical treatment at Qingdao University Affiliated Hospital. B-ultrasound revealed an enhanced echo in the lower uterine segment, measuring approximately 5.6×4.8×3.8 cm, raising suspicion of endometrial carcinoma (**Figure 1**). Diagnostic endometrial biopsy subsequently confirmed adenocarcinoma, favoring endometrioid subtype. Serum tumor markers were as follows: Alpha-fetoprotein (AFP) 15.480 ng/mL (reference range: 0–7 ng/mL), carcinoembryonic antigen (CEA) 8.070 ng/mL (reference range: 0–5 ng/mL), carbohydrate antigen 199 (CA199) 98.280 U/mL (reference range: 0–30 U/mL), and carbohydrate antigen 125 (CA125) 8.080 U/mL (reference range: 0–25 U/mL). Whole-body PET/CT demonstrated an ill-defined hypermetabolic soft-tissue nodule (SUVmax ≈13.8) in the uterine corpus-cervix junction and an intracavitary protruding nodule (SUVmax ≈9.3) in the fundus, with no enlarged lymph nodes in the bilateral iliac/inguinal regions, consistent with uterine malignancy (**Figure 2**). Following an initial diagnosis of endometrial malignancy, the patient was admitted to our gynecology department on February 16, 2024, for surgery. Following admission, a comprehensive medical history was documented. The patient is G3P2A1. Fourteen years ago, she underwent surgical treatment for rectal cancer, followed by adjuvant chemoradiotherapy. Regular postoperative surveillance revealed no signs of recurrence. On February 20, 2024, the patient underwent laparoscopic exploration Laparoscopy revealed dense adhesions between the rectum/sigmoid colon and left pelvic wall, with fibrosis impeding vascular dissection. Frozen section pathology indicated moderately-to-poorly differentiated adenocarcinoma with necrosis and calcification, infiltrating nearly the full myometrial thickness. Consequently, we performed laparoscopic extrafascial total hysterectomy, bilateral adnexectomy, right pelvic lymph node dissection, para-aortic lymph node biopsy, and pelvic adhesion lysis.

**Figure 3:**
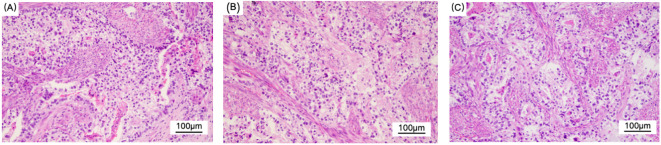
Figure **(A–C)** show the light microscopic appearance of YST. Hematoxylin and eosin (H&E), original magnification × 200.The main type is adenocarcinoma. Some tumor cells have clear cytoplasm, and nuclear/proximal nuclear vacuoles can be observed in figure **(B)**. They are arranged in an adenoid or sieve-like pattern, and mitotic figures are common. Considering the morphology and immunohistochemical results, it is considered a mixed endometrial-like carcinoma. Some of the tumors are suspected to have differentiated into yolk sac tumors.

**Figure 4:**
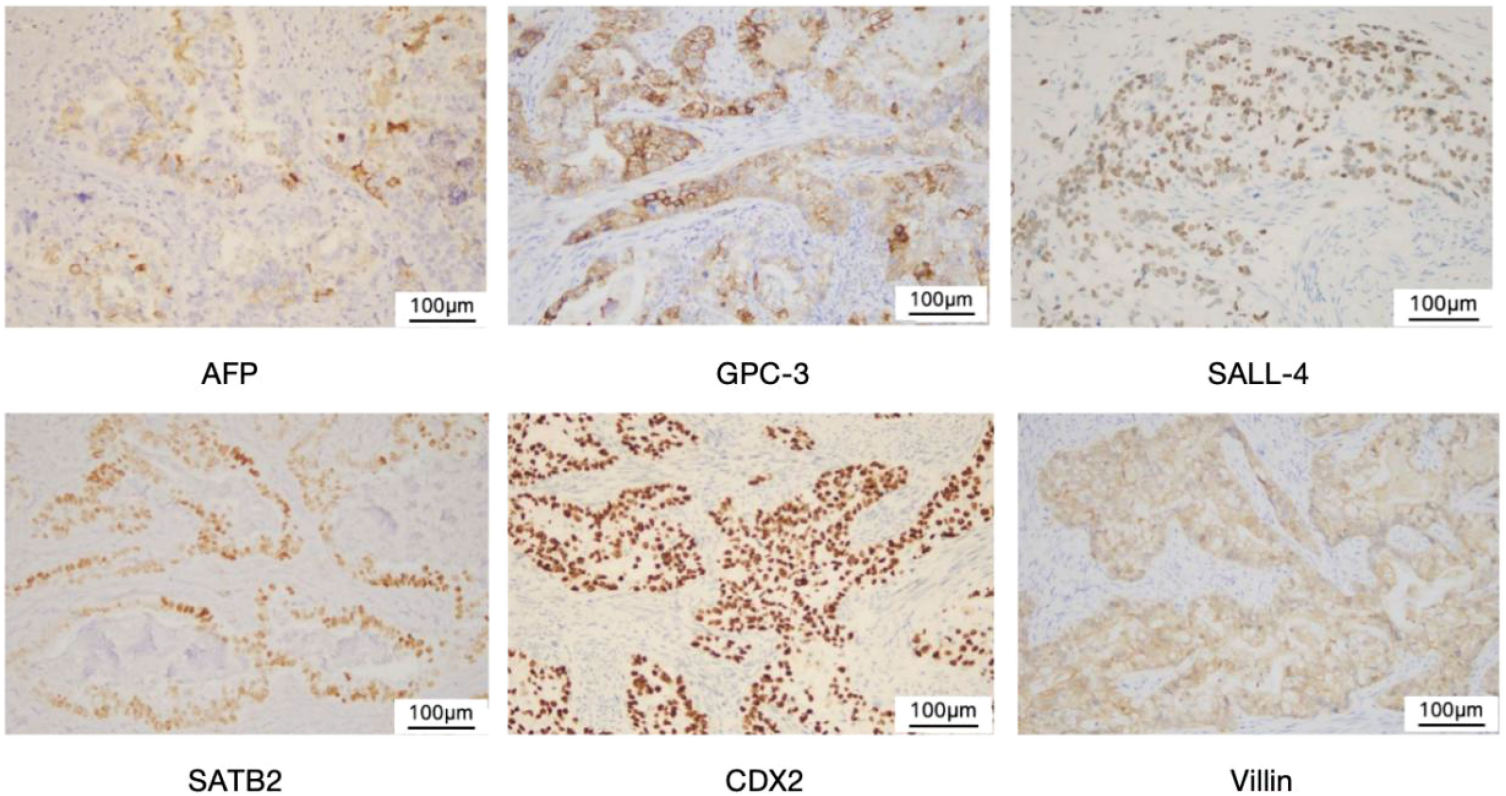
Immunohistochemical staining of YST.Yolk sac tumor (× 200). Immunohistochemical staining showed positive for AFP,GPC-3, SALL-4, SATB2,CDX2, and Villin, respectively.

The size of the removed uterus was approximately 8 cm x 8 cm x 2 cm. A rough area measuring 6 cm x 5.5 cm was found in the uterine cavity, characterized by tough gray matter that visibly invaded the entire layer. Additionally, a mass measuring 2.5 cm x 2.1 cm was identified in the uterine cavity, exhibiting gray, white, and red sections with a slightly tough consistency. The tumor invaded the right pelvic peritoneum but did not involve the lymph nodes. According to the FIGO (International Federation of Obstetrics and Gynecology) staging system, this case is classified as Stage IIIB. Microscopically, the main type of cancer was adenocarcinoma. In some areas, the cytoplasm of the tumor cells was transparent, and nuclear/paranuclear vacuoles could be observed, presenting an adenoid or sieve-like arrangement. Nuclear mitotic figures were common (**Figure 3**). Based on the morphology and immunohistochemical results, it was considered a mixed endometrial-like carcinoma. Some of the tumors were suspected to have differentiated into yolk sac tumors, invading the entire layer of the uterine body muscle wall. Many vascular tumor thrombi could be seen, invading the cervical canal fibromuscular layer (about 1/2 layer), involving the right pelvic peritoneum and the right ovarian vessels, but not the bilateral parametrial tissues or lymph nodes. Immunohistochemistry showed AFP (foci+), SALL4 (+), GPC3 (+), SATB2 (+), CDX2 (+), Villin (+), Pax-8 (-), Vimentin (-), CK7 (in a small part +), CK20 (+), CD10 (+) (**Figure 4**).

Genetic testing revealed: 1) TP53 C275Y mutation (17.5% VAF); 2) Endometrial carcinoma molecular subtype: copy-number high; 3) PD-L1 CPS = 0.3 (negative); MSI = 0.041 (MSS); TMB = 3.110 Muts/Mb (TMB-low).Postoperatively, the patient received three cycles of BEP chemotherapy: bleomycin (24 mg; 15 U/m², D1-2), etoposide (162 mg; 100 mg/m², D1-3), cisplatin (48 mg; 30 mg/m², D1-3). Due to significant adverse reactions, subsequent doses were reduced to: bleomycin (12 U/m², D1-2), etoposide (130 mg; D1-3), cisplatin (40 mg; 25 mg/m², D1-3). From 2024-05-29, she underwent 23-fraction vaginal brachytherapy (4600 cGy total, 200 cGy/fraction) with cisplatin radiosensitization (30 mg/m², D1) on 2024-06–01 and 2024-06-19, which was well-tolerated. An additional BEP cycle was administered on 2024-08-05. Serum AFP normalized before the first chemotherapy cycle and remained normal during treatment.

**Figure 5:**
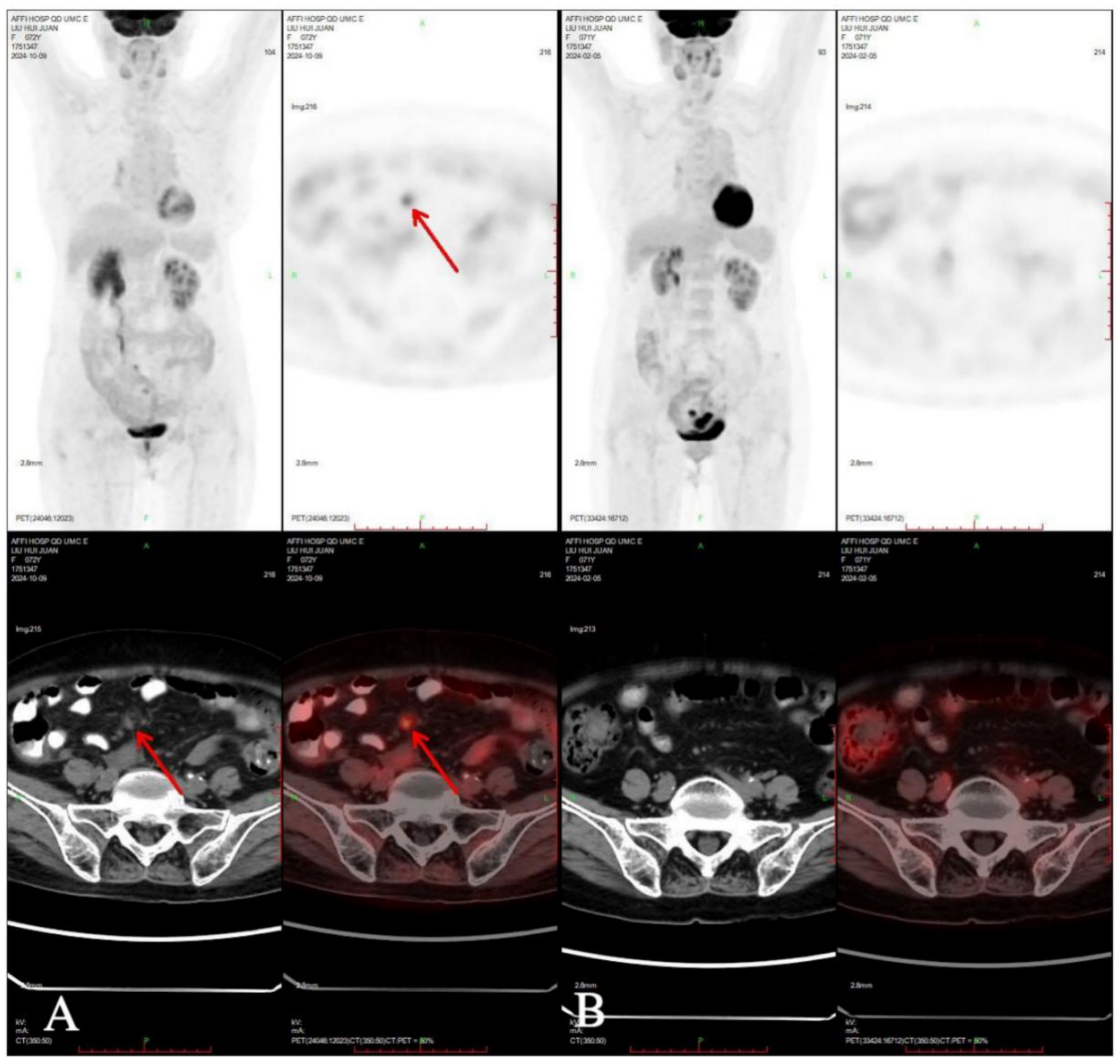
Compared PET-CT **(A)** in 2024-10-09 with PET-CT **(B)** in 2024-02-05, new lesions were found in the omentum.

**Figure 6:**
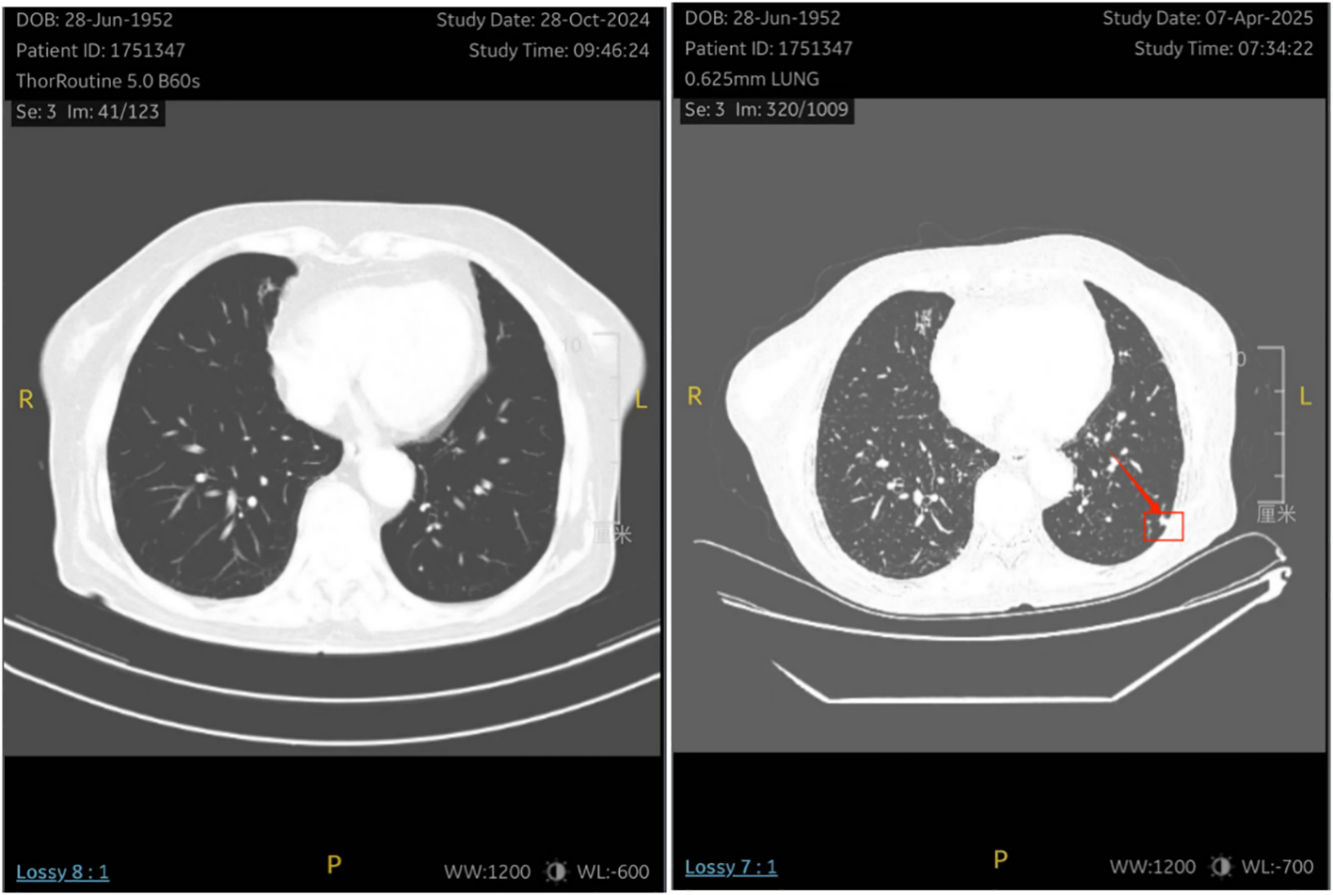
The patient underwent a re-examination of the chest CT on April 7, 2025, which revealed multiple small nodules in the lungs . It is considered that there might be metastatic tumors.

One month after chemotherapy, the patient developed dysuria. Ultrasound showed right hydronephrosis, and CT suggested possible metastatic nodules in the left pelvic peritoneum. PET/CT revealed a new hypermetabolic soft-tissue lesion (SUVmax ≈4.3) in the mesentery (**Figure 5**), indicating recurrence. Serum AFP was measured at 3.16 ng/mL (reference range 0–7 ng/mL). Given these examination results, the possibility of metastasis and recurrence was considered. The patient requested continued observation without treatment. On April 7, 2025, chest CT detected multiple pulmonary nodules suggestive of metastasis (**Figure 6**), though AFP remained normal. Multidisciplinary consultation recommended paclitaxel (240 mg/m²) + carboplatin (AUC = 5) + pembrolizumab (2 mg/kg).

## Discussion

**Figure 7:**
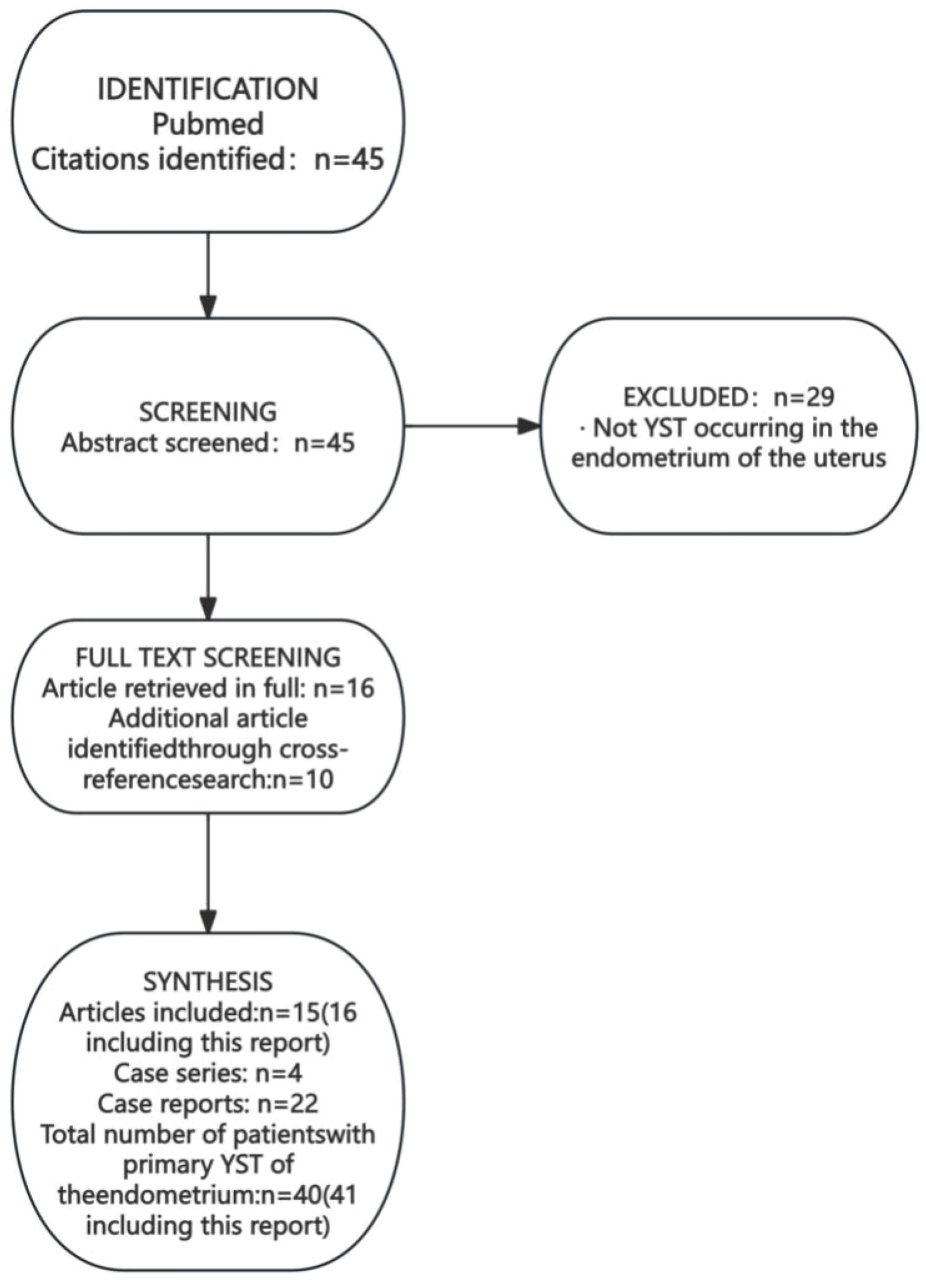
Literature search process for cases of primary endometrial yolk sac tumor.

We conducted a systematic literature search in PubMed in October 2024 using the search string: ((“yolk sac tumor*”[tiab] OR “yolk sac tumor*”[tiab] OR “endodermal sinus tumor*”[tiab] OR “endodermal sinus tumour*”[tiab] OR “EST”[tiab] OR “YST”[tiab]) AND (“endometr*”[tiab] OR “uter*”[tiab] OR “uterine corpus”[tiab]) AND (“primary”[tiab] OR “primitive”[tiab] OR “*de novo*”[tiab])) AND (“case report”[pt] OR “case series”[pt] OR “case”[tiab] OR “report”[tiab] OR “review”[tiab]). A total of 45 articles were retrieved. After excluding irrelevant articles and supplementing based on cross-referenced literature, 37 cases of primary uterine endometrial YST (26 publications) were included (**Figure 7**) (6–31). Among them, there were 21 cases of simple uterine endometrial primary yolk sac tumor and 19 cases of mixed uterine endometrial primary yolk sac tumor. We reviewed and summarized the symptoms, pathological features, treatment modalities, and prognosis of these 37 known cases of primary endometrial yolk sac tumor (**Table 1**). The first case of primary endometrial yolk sac tumor was reported by Ishiguro T in 1980 (6). In 1996, Shokeir MO reported the first case of a uterine malignant Mullerian mixed tumor containing yolk sac tumor components (7), which was subsequently recognized as the first case of mixed primary endometrial yolk sac tumor. In other reported cases, the tumor components were mixed with endometrial adenocarcinoma (4/19), serous carcinoma (3/19), undifferentiated carcinoma (3/19), adenocarcinoma (3/19), clear cell carcinoma (3/19), carcinosarcoma (2/19), embryonal carcinoma (1/19), High-grade cancer (1/19) and immature teratoma (1/19). The patient in the present case had a primary endometrial yolk sac tumor complicated by endometrioid carcinoma.

**Table 1:** Summary of clinicopathologic features of primary endometrial YSTs.

Author	Year of public	Age	Symptom	Associated component	Initial diagnosis	FIGO stage	Metastasis site	Laboratory report	Immunohistoch-emical	Surgerys	Chemo- therapy	Radiotherapy	Follow up
Pileri (6)	1980	28	AVB	None	NA	IB	None	AFP 380ng/ml	AFP(+)	TAH+BSO	VAC	No	REC 2;DOD 8
Ohta (9)	1988	27	AVB,IM	None	YST	IA	None	AFP 1580ng/ml	AFP(+)	TAH+BSO+OMT	VAC	No	NED>14
Clement (10)	1988	24	AVB	None	NA	IV	Ovary	AFP 3600ng/ml	AFP(+),AAT(+), CEA(+)	CISH+BSO	VAC	YES	REC,DOD 24
Joseph (11)	1990	42	AVB	None	YST	IA	None	AFP18530ng/ml	AFP(+), AAT(+), CK(+),PLAP(+)	TAH+BSO	PVB	No	NED>24
Spatz (12)	1998	49	AVB	None	YST	IVB	None	AFP normal	AFP(+)	TAH+BSO+PLND	NO	45 Gy on the pelvis	NED>28
Wang C (13)	2011	29	AVB	None	YST	II	None	AFP 3593.4 ng/ml	NA	Modified hysterectomy+LSO+PLND+PALND	Etoposide + carboplatin + bleomycin	No	NED>39
Rossi R (14)	2011	30	AVB	None	GCT	II	None	AFP1.762 ng/ml	AFP(+)	TAH	BEP	No	NED>72
Abhilasha (15)	2014	31	AVB	None	NA	IA	None	AFP 242.3 IU/ml	AFP(+),CK(+), PLAP(+),CD 117(+)	TAH+BSO+PLND+PALND+OMT	BEP	No	NED >24
Qzler (16)	2015	44	AVB	None	NA	IB	None	AFP 27522ng/ml	NA	TAH+BSO+PLND+PALND+OMT	BEP	No	NED>6
Qzler (16)	2015	57	Abd pain	None	NA	IVB	Lung,ovary, liver	AFP 29214ng/ml	NA	TAH+BSO+PLND+PALND+OMT	BEP	No	DOD<2
Damato (17)	2016	63	Postmenopausal bleeding	None	Metastatic colorectal carcinoma	IVB	Liver,lung	AFP 244.6IU/ml(PO)	AFP(+),GPC3(+),SALL4(+),Villin(+), CDX2(+), Hep Par1(+), CK20(+),CEA(+)	TAH+BSO+PLND+PALND+OMT	BEP	No	REC,6
Li JK (18)	2017	38	Menorrhea	None	NA	IIIC	Omentum	AFP 37.4 ng/ml(PO)	AFP(+),CEA(+), SALL4(+), CK18(+),CK56(+)	TAH+BSO+PLND+PALND+OMT	BEP	No	NED>24
Ravishankar (8)	2017	59	AVB,uterine mass	None	Endometrial adenocarcinoma	IB	NA	NA	AFP(+),GPC3(+)SALL4(+), CDX2(+)	NA	YES	No	LTF
Ravishankar (8)	2017	68	AVB,uterine mass	None	Metastatic colorectal carcinoma	IV	NA	NA	AFP(+),GPC3(+), SALL4(+), CK20(+)	YES	YES	No	DOD 14
Ravishankar (8)	2017	61	AVB	None	Metastatic colorectal carcinoma	IA	NA	NA	AFP (–),CK20(+), CK7(+),CDX2(+), SALL4(+)	YES	YES	No	AWD8
Lu T (19)	2019	27	AVB	None	YST	IA	NA	AFP 800 ng/ml	AFP(+),SALL4(+), CK(+)	TAH+BSO+PLND+PALND+OMT	DC	No	NED> 14
Lin SW (20)	2019	68	Postmenopausal bleeding	None	Serous carcinoma	II	None	AFP 133.4ng/ml(PO)	AFP (+)	TAH+BSO+PLND+PALND+OMT	BEP	No	NED>6
Ge H (21)	2021	43	AVB,Abd pain	None	EC	IA	None	AFP 1465 µg/ml	AFP(+),SALL 4(+), GPC-3(+),AE1/AE3(+),PAX 8(+)	TAH+Bilateral salpingectomy+ Bilateral ovarian biopsy +PLND+PALND+OMT	BEP	No	NED >15
Sinha (22)	2021	73	Abd pain	None	EC	IIIA	Ovary	AFP10.5 ng/ml(PO)	AFP(+),GPC3(+), SALL 4(+)	TAH+BSO+PLND+PALND+Omental biopsy	BP	No	NED>15
Cheng X (23)	2021	35	AVB, prolonged menstruation	None	NA	IA	None	AFP 9152 ng/ml	AFP(+),SALL4(+), AE1/AE3(+),CD117(+),Ki-67(80%+), Vim(+)	TAH+BSO+PLND+PALND+OMT	BEP	No	NED>21
Liu R (24)	2024	42	AVB	None	YST	IIIA	None	AFP1210 ng/ml	AFP(+),SALL4(+), p16(+),EMA(+),	TAH+BSO+PLND	BEP	No	NED>13
Shokeir (7)	1996	64	Abd	mixed Müllerian tumor	NA	IVB	NA	AFP15918ng/ml	NA	NA	NA	No	DOD,2.5
Patsner (25)	2001	59	Postmenopausal bleeding	EC	Endometrial adenocarcinoma	IVB	Liver, diaphragm	AFP 27670 ng/ml, CA-125 250 U/ml	AFP(+), CK(+),PLAP(+)	TAH+BSO+PLND+OMT	BEP EP	21 Gy by vaginal brachytherapy	REC,16;AWD>16
Oguri (26)	2006	65	Watery discharge	carcinosarcoma	Endometrial adenocarcinoma	IIIC	Ln	AFP 2360 ng/ml, CA 199 50 U/ml, CEA 110 U/ml	AFP(+), AE1/AE3(+), CK7(+),EMA(+)	MRH+BSO+PLND	TP	No	NA
Ji M (27)	2013	28	AVB	EC	Endometrial adenocarcinoma	IV	Peritoneum,Omentum	AFP 1522 ng/ml, β-hCG 518.9 mIU/ml, CA 125 129 U/ml	AFP(+), AE1/AE3(+), EMA(+), CA125(+),CK7(+)	TAH+BSO+OMT+PLND+appendectomy+ partial resection of the sigmoid colon with anastomosis	PTX,ADM,DDP,CBDCA,MTX,Act-D,VP-16,BLM,pingyangmycin,VCR,FUDR,oxaliplatin,CPA	No	REC,2;AWD,10
Hu Y (28)	2016	36	AVB	CCC	CCC	IIIA	None	AFP 4597 ng/ml	AFP(+), GPC3(+),CK(+), Calretinin(+), PLAP(+)	TAH+BSO+PLND+PALND+OMT	Docetaxel+nedaplatin	No	NED >12
Ravishankar (8)	2017	55	AVB	Complex hyperplasia	Endometrial adenocarcinoma	II	NA	NA	AFP (–),CK20(+), CDX2(+)	YES	YES	YES	DOD16
Ravishankar (8)	2017	77	AVB,uterine mass	Endometrial adenocarcinoma, Undifferentiated carcinoma	MMMT	IIIC	NA	NA	AFP(-),GPC3(+),SALL4(+),PAX-8(+)	NA	NA	NA	LTF
Ravishankar (8)	2017	64	AVB	adenocarcinoma,NOS	Undifferentiated carcinoma	IIIA	NA	NA	CK7(+),CDX2(+),AFP(+),GPC3(+),SALL4(+)	YES	YES	YES	DOD 23
Ravishankar (8)	2017	87	AVB	adenocarcinoma,NOS	Endometrial adenocarcinoma	II	NA	NA	AFP(+), GPC3(+), SALL4(+)	YES	YES	No	AWD7
Ravishankar (8)	2017	63	AVB	MMMT	MMMT	IIIC	NA	NA	CDX2(+), Villin(+), SALL4(+)	YES	YES	YES	NED5
Ravishankar (8)	2017	62	AVB	Serous carcinoma	Serous carcinoma	IB	NA	NA	AFP (–),CK7(+), GPC3(+),SALL4(+), PAX-8(+)	YES	YES	No	AWD30
Ravishankar (8)	2017	77	AVB	Serous, Clear cell, Undifferentiated carcinoma	Serous carcinoma	IIIC	NA	NA	AFP (–),CDX2(+), GPC3(+),SALL4(+), PAX-8(+)	YES	YES	No	AWD17
Song L (29)	2019	38	AVB,menorrhea	CCC	Carcinosarcoma	IVB	None	CA125 58.5 U/ml,AFP Normal	AFP(+),SALL4(+), GPC3(+),CK18(+), CD56(+),CD15(+), p16(+),CEA(+)	TAH+BSO+PLND+PALND+OMT+Appendectomy	BEP	No	NED>24
Zhang H (30)	2020	65	Postmenopausal bleeding	embryonal carcinoma,immature teratoma	EC	1A	None	AFP 359 ng/ml	AFP(+),OCT 3/4(+), AE1/AE3(+), GFAP(+),Ki-67(+)	TAH+BSO	BEP	No	NED 15
Mills (31)	2024	74	Postmenopausal bleeding	Carcinosarcoma	YST	IB	NA	AFP10024 ng/ml	AFP(+),GPC3(+), SALL4(+)	YES	NA	NA	NA
Mills (31)	2024	82	NA	Highgrade carcinoma	Highgrade carcinoma	IB	Lung	NA	AFP(+),GPC3(+), SALL4(+),CDX2(+),CK20(+)	YES	NA	NA	REC,8
Mills (31)	2024	70	NA	SC	SC	IB	None	NA	AFP(-),SALL 4(+), GPC-3(+)	YES	NA	NA	NED>34
Mills (31)	2024	59	NA	UDC	UDC	IA	None	NA	SALL 4(+), GPC-3(+)	YES	NA	NA	NED>48
Zicheng C	–	71	Postmenopausal bleeding	EC	EC	IIIB	Omentum,lung	AFP 15.480ng/ml CEA 8.070ng/ml CA199 98.280U/ml	AFP(+),SALL4(+),GPC3(+),SATB2(+)CDX2(+),Villin(+),CK7(+),CK20(+),CD10(+)	TAH+BSO+PLND	BEP+TC	CTV4600 cGy/23f by vaginal brachytherapy	REC,8;AWD>20

ref, references; AVB, abnormal vaginal bleeding; Abd, abdominal; PO, postoperatively; EC, endometrioid adenocarcinoma; SC, serous carcinoma; UDC, undifferentiated carcinoma; AC, adenocarcinoma; MMMT, malignant mixed mullerian tumor; CCC, clear cell carcinoma; CISH, classical intrafascial supracervical hysterectomy; BSO, bilateral salpingo-oophorectomy; TAH, total abdominal hysterectomy; OMT, omentectomy; PLND, pelvic lymph node dissection; PALND, para-aortic lymph node dissection; MRH, modified radical hysterectomy; REC, recurrence; AWD, alive with disease; DOD, died of disease; LTF, lost to follow-up; NED, no evidence sof disease; NA, not available.

Primary endometrial YST typically presents with irregular vaginal bleeding as an early symptom; some patients also experience abdominal pain, vaginal discharge, or menstrual abnormalities. The mean age at diagnosis was 53.78 years (range: 24-87). Patients with pure endometrial YST were significantly younger than those with mixed tumors (mean age 44.67 *vs*. 61.37 years; p = 0.005). Age >50 years was associated with higher mortality risk (p = 0.016). FIGO staging was available for 41 patients: Stage I (n=16), II (n=4), III (n=11), and IV (n=9). Pure YSTs were diagnosed at earlier stages (Stage I: 47.62% [10/21], II: 14.29% [3/21], III: 14.29% [3/21], IV: 23.81% [5/21]), while mixed YSTs presented at more advanced stages (Stage I: 15.00% [6/20], II: 10.00% [2/20], III: 40.00% [8/20], IV: 20.00% [4/20]), though this difference was not statistically significant (p=0.40).

Yolk sac tumor (YST) is an alpha-fetoprotein (AFP)-secreting germ cell malignancy. Serum AFP levels have high specificity in the diagnosis of yolk sac tumors, but there is no absolute threshold. When the serum AFP index exceeds 10 times the upper limit of normal (ULN), it is a strong indicator supporting the diagnosis (for example, when ULN = 10 ng/mL, AFP > 100 ng/mL). Most patients with yolk sac tumors have AFP levels above 1,000 ng/mL (32). However, the threshold of serum AFP levels for yolk sac tumors in different locations varies at present, and its diagnostic value needs to be comprehensively judged in combination with clinical manifestations, pathology and other examinations. Serum AFP measurement is valuable for diagnosis, monitoring treatment response, and detecting metastasis or recurrence post-therapy. Apart from two patients whose AFP levels were within the normal range before and after surgery (7, 29), the AFP levels of other patients were significantly elevated before or after surgery. Other tumor markers, such as CA199, CA125, and CEA, generally remain within the normal range but have been elevated in some cases (25–27, 29). Mingliang Ji reported a case of elevated β-hCG (β-hCG = 518.9 mIU/mL) in a patient with endometrial yolk sac tumor, which was considered to be associated with a trophoblastic tumor (27). In our case, AFP was mildly elevated (AFP = 15.47 ng/mL), along with elevated CEA (CEA = 8.070 ng/mL, reference range: 0–5 ng/mL) and CA199 (CA199 = 98.280 U/mL, reference range: 0–30 U/mL), which may be related to the presence of other malignant tumor components.

The mechanism of extragonadal YST remains controversial, but four possible mechanisms have been proposed: (1) Extragonadal YST may originate from ectopic or primordial germ cells that remain along the midline of the human spine during embryogenesis. These cells may remain in the basal layer of the endometrium for long periods, and thus, extragonadal YST is often associated with other germ cell tumors. (2) It may be caused by residual fetal tissue after an incomplete abortion. Sobis and Vandeputtr (33), Sakashita et al. (34), and others (35) have reported that fetal resection can induce intrauterine sinus tumors in rats and mice, and it is possible that YST can develop from tissue remaining after spontaneous abortion in humans. (3) Primary gonadal occult germ cell tumor metastasis. (4) Extragonadal YST may also result from abnormal differentiation of somatic cells, which may explain some mixed malignant cell tumors. Current evidence suggests that YST originating from aberrant somatic differentiation predominantly affects postmenopausal women. McNamee et al. proposed that YST in adults may develop secondarily within overgrown epithelial tumors. The YST component could arise from these epithelial precursors via neometaplasia or dedifferentiation processes. Consequently, they designated such tumors as somatic-type YST (36).

Among the 22 reported cases of pure endometrial YST, 14 patients underwent preoperative diagnostic curettage. Pathological analysis confirmed YST diagnosis in 7 of these patients. Notably, diagnostic curettage in three patients suggested metastatic colorectal adenocarcinoma, a potential misdiagnosis possibly attributable to the tendency of YST to differentiate toward endodermal derivatives, such as intestinal tissue. In mixed endometrial YST, the complex histological composition often impedes the identification of the YST component through preoperative curettage. Indeed, among the 19 previously reported cases, no YST component was detected preoperatively. In the case we present, preoperative curettage resulted in a diagnosis of endometrial adenocarcinoma. The diagnosis of yolk sac tumor relies on pathological examination, and its histological patterns are notably more diverse than those of other germ cell tumors. Characteristic histological features of YST include (37): (1) loose reticular structure, (2) Schiller-Duval body (S-D body), (3) transparent corpuscles, (4) acinar, glandular, or adenoid structures of varying sizes and shapes, and (5) papillary and cystic structures. When considering SDYST, mere morphology is not sufficient for its diagnosis. Fadare’s morphological review of 626 endometrial carcinomas identified 5 cases (0.8%) exhibiting potential YST features; among these, 3 (0.5%) were confirmed as true somatic-type YST by supportive IHC (38). Consequently, histological variability and the propensity of YST to mimic somatic carcinomas can complicate diagnosis, particularly when the tumor arises in atypical locations (e.g., extragonadal) or outside the characteristic age group. Immunohistochemical analysis is crucial in these scenarios. Although AFP demonstrates high specificity and was historically considered vital for histological confirmation of YST, its diagnostic utility is limited. Focal AFP immunopositivity can occur in epithelial carcinomas, embryonal carcinomas, and immature teratomas (39). Moreover, not all YSTs exhibit positive AFP immunostaining (40), as evidenced by 5 reported AFP-negative cases. This has prompted exploration of additional diagnostic markers.SALL4, a pluripotency marker associated with germ cell differentiation, is expressed in all germ cell tumors except choriocarcinoma. It is therefore highly valuable for confirming the presence of a germ cell component but exhibits lower specificity for YST specifically (41). Glypican-3 (GPC-3) is a more sensitive marker for YST and may outperform AFP in highlighting glandular and hepatoid patterns. However, GPC-3 expression is also found in other somatic tumors, particularly clear cell carcinoma, which shares histological overlap with YST and can cause diagnostic confusion (42). When used in combination with GPC-3 and AFP, SALL4 serves as a useful diagnostic marker (43).Mills AM proposed an IHC-based diagnostic algorithm for somatic-type YST: appropriate morphology combined with coexpression of at least two YST markers (SALL4, Glypican-3, AFP), with at least one marker showing expression in ≥70% of cells within areas demonstrating morphological features of YST differentiation. Given the high specificity of AFP, its presence in any proportion of cells can permit less extensive staining for SALL4 and Glypican-3 (31).Due to its potential for differentiation toward endodermal derivatives (e.g., intestinal tissue), YST may express markers commonly associated with somatic tumors, such as cytokeratins 7 and 20, CDX2, and villin (44). Villin, expressed throughout endodermal development, was suggested by Nogales et al. (2014) for inclusion in the diagnostic IHC panel for YST (45).

Follow-up information was available for 37 out of 41 patients. The mean follow-up duration was 18.14 months (range: 2–72 months). Disease-free survival was observed in 54.05% (20/37) of patients. Adverse outcomes included recurrence in 18.92% (7/37) and disease-related death in 21.62% (8/37) within 2.5 to 24 months. Persistent disease was present in 21.62% (8/37) of patients at their last follow-up. The incidence of adverse outcomes (recurrence or death) showed a significant association with FIGO stage (p = 0.012). Adverse outcomes occurred in 77.78% (7/9) of FIGO stage IV patients, 33.33% of stage III patients, and 15.00% and 20.00% of stage I and II patients, respectively. Among the 20 patients with pure primary endometrial YST and available follow-up, 25% (5/20) experienced adverse outcomes. For the 17 patients with mixed primary endometrial YST and follow-up, the adverse outcome rate was 47.06% (8/17). Although mixed tumors suggested a trend toward worse prognosis, the difference did not reach statistical significance (P = 0.207).

Due to the limited number of cases of primary endometrial YST, there is no consensus on treatment. Currently, surgery and postoperative adjuvant therapy are the primary treatment modalities. Of the 41 known cases, 38 patients underwent surgical treatment. Details of the surgical approach were available for 25 patients. All of these patients had a total hysterectomy, 88% underwent bilateral salpingo-oophorectomy (22/25). Two patients preserved bilateral ovaries due to fertility concerns, and one patient had her right ovary preserved. Left salpingo-oophorectomy, pelvic lymphadenectomy, and/or anterior sacral lymphadenectomy were performed in 82% of patients (18/25). Fourteen patients also underwent greater omentectomy, one patient had appendectomy, and two patients had both procedures. Nearly half of the patients (41.46%, 17/41) were women of reproductive age. Therefore, whether endocrine function should be preserved in young women of reproductive age diagnosed with primary endometrial YST warrants further investigation. Changyu Wang et al. reported a 29-year-old woman diagnosed via dilation and curettage pathology with primary endometrial YST (13). She underwent modified hysterectomy, left salpingo-oophorectomy, and pelvic and para-aortic lymphadenectomy, followed by adjuvant chemotherapy with bleomycin, etoposide, and carboplatin. The right adnexa was preserved to maintain endocrine function. Follow-up examinations at 39 months post-diagnosis showed no abnormalities. Roberto et al. reported a 30-year-old woman with primary endometrial YST who underwent simple total hysterectomy with bilateral adnexal preservation; pelvic and para-aortic lymph nodes were not resected. She received three cycles of bleomycin, etoposide, and cisplatin (BEP) postoperatively and remained disease-free for over 6 years after treatment (14). Consequently, preserving both ovaries in young women under close monitoring appears feasible. However, more case data are needed to support this approach, as ovarian metastasis occurred in 3 of 37 reported cases with follow-up after treatment. Besides ovarian metastasis, among 24 cases with available follow-up information, metastases were also observed in the liver (3/24), lungs (2/24), omentum (2/24), lymph nodes (2/24), peritoneum (1/24), and diaphragm (1/24).

There is similarly no standardized chemotherapy regimen for primary endometrial YST. For ovarian germ cell tumors (GCT), regimens such as BEP (bleomycin, etoposide, cisplatin), BVP (bleomycin, vincristine, cisplatin), or VAC (vincristine, actinomycin D, cyclophosphamide) are recommended. Changyu Wang et al. propose that bleomycin, etoposide, and cisplatin is the preferred first-line adjuvant therapy for both ovarian and endometrial YST (13). According to the 1994 Gynecologic Oncology Group (GOG) study (GOG protocol 78), the standard recommended treatment for ovarian GCT is PEB (cisplatin, etoposide, bleomycin). This study demonstrated that three courses of adjuvant BEP following complete resection of ovarian GCT nearly always prevented recurrence (46). Among the 41 current patients, 33 received chemotherapy, with details available for 24: 14 received BEP; one patient received BP (cisplatin, bleomycin) due to abnormal pulmonary function precluding bleomycin use (28); one received a combination of etoposide, bleomycin, and carboplatin; VAC was used in 12.5% (3/24); PVB (cisplatin, vinblastine, bleomycin) in 4.16% (1/24); and other regimens in 20.8% (5/24). Research by Wang X et al. showed that BEP chemotherapy combined with the anti-angiogenic targeted agent bevacizumab and immunotherapy (tislelizumab) achieved good efficacy in treating postoperative pelvic and lymph node metastases, offering a new approach for maintenance therapy after surgery for primary endometrial YST (47).

The literature reports 6 cases of postoperative adjuvant radiotherapy: 1 case of a 24-year-old patient with endometrial YST, who received 6 courses of VAC chemotherapy after surgery, and died of the tumor 2 years after the onset of the disease (10). Spatz (12) et al. reported a case of a 49-year-old patient who underwent hysterectomy and bilateral adnexectomy, and only pelvic external irradiation was performed after surgery. There was no tumor recurrence after 28 months of follow-up. A 59-year-old postmenopausal woman with endometrial adenocarcinoma with YST components received vaginal internal radiotherapy, 21 Gy/1w, and the tumor recurred 1 month after the surgery (25). Ravishankar (8) et al. reported 3 patients who received adjuvant radiotherapy and concurrent chemoradiotherapy after surgery. One patient survived without tumor for 5 months, and 2 patients died of disease progression. Their survival periods were 16 months and 23 months respectively. In this case, radiotherapy and concurrent chemoradiotherapy were also applied after surgery, but metastasis and recurrence occurred in the omentum 2 months after the end of chemotherapy. The prognosis of these 7 patients varied greatly. Whether postoperative adjuvant radiotherapy can improve the prognosis of patients needs further study.

## Shortcomings and prospects

The reported cases of endometrial YST are very limited, whether it is simple YST or SDYST. Therefore, doctors have limited experience in diagnosing and treating such diseases. Fadare’s re-examination of 626 cases of endometrial malignancies revealed that there were 3 cases of missed diagnosis of SDYST (37). Therefore, we cannot help but suspect that there were missed diagnoses in previous cases of diagnosing endometrial malignancies. This also requires pathologists to improve their diagnostic ability for such tumors. Fadare proposed a diagnostic model combining pathological features, AFP, GPC-3, and SALL4, which provides new insights for the diagnosis of SDYST. More cases are still needed to increase the clinical treatment experience for this disease. Current issues discussed regarding the treatment plan for this disease include but are not limited to: 1. Treatment plans for young patients with simple endometrial YST to preserve fertility; 2. Whether the omentum should be removed during surgery; 3. Whether patients with endometrial YST who meet the radiotherapy indications for endometrial cancer can benefit from radiotherapy. 4. For SDYST, whether the chemotherapy plan needs to be appropriately adjusted according to the type of somatic cell tumors and the proportion of yolk sac tumors it is combined with. 5. Treatment plans after recurrence.

## Conclusion

Primary endometrial yolk sac tumor is an exceedingly rare malignant germ cell tumor. Early symptoms typically include vaginal bleeding and abdominal pain, often accompanied by elevated serum AFP levels. Schiller-Duval bodies are a characteristic histopathological feature, while immunohistochemical markers (AFP, GPC-3, SALL4, Villin) aid in distinguishing it from other malignancies. The mean follow-up duration for primary endometrial YST was 18.14 months (range: 2–72 months); 54.05% (20/37) of patients showed no evidence of disease during follow-up. Advanced FIGO stage correlated with higher recurrence and mortality rates. Patients aged >50 years exhibited increased mortality risk. The prognosis of mixed (SDYST) primary endometrial YST appears poorer. The currently most primary treatment is radical surgery followed by adjuvant BEP chemotherapy (bleomycin, etoposide, cisplatin). Whether radiotherapy improves prognosis remains to be determined through further investigation.

## Data Availability

The original contributions presented in the study are included in the article/supplementary material. Further inquiries can be directed to the corresponding authors.
